# Becoming a Caregiver: The Role of the Environment in the Process of Children Becoming Responsible for Others

**DOI:** 10.3390/bs15010049

**Published:** 2025-01-07

**Authors:** Carolina Remorini

**Affiliations:** Departament d’ Antropologia Social i Cultural, AFIN Research Group & Outreach Centre, Universitat Autònoma de Barcelona, 08193 Bellaterra, Spain; carolina.remorini@uab.cat

**Keywords:** child development, learning, socio-emotional skills, environment, indigenous, anthropology

## Abstract

This article aims to illustrate the pivotal role that the environment plays in early child development (ECD), drawing upon data derived from the ethnographic research on children’s daily routines. Participant observation and in-depth interviews were conducted over the course of several fieldwork periods (2013–2018). The concept of “mutual raising” is employed to examine the daily interactions between middle-aged children and their domestic animals. To gain an insight into how children develop sophisticated and ecologically relevant skills to become autonomous and responsible for the care of others within the context of environmental interdependence, we present and analyze a cultural practice that is salient to the identity and way of life in rural communities of the Andean region in South America: becoming a shepherd. Considering the aforementioned findings, it can be posited that being able to care for others represents a significant developmental milestone. This discussion highlights the limitations of the dominant theoretical and disciplinary lens through which ECD is currently studied, those so-called W.E.I.R.D societies. Consequently, it is imperative to advocate for an integrative and transdisciplinary framework for ECD studies that incorporates anthropological evidence and the cultural experiences of children and families who have been historically marginalized by dominant ECD models.

## 1. Introduction

The science of early childhood (ECD) primarily evolves through the research conducted on populations with distinctive socio-economic and cultural characteristics. These populations are often designated as “W.E.I.R.D” (Western, Educated, Industrialized, Rich, and Democratic) societies or, more recently, as the “minority world”. The concept of majority–minority-world opposition, coined by Alam (2008), emerged to explain why certain developmental and health models from societies comprising only about 12% of the world’s population were considered “universal” for humanity. This perspective overlooks the fact that most of the world’s societies have distinct ways of life and practices, often deemed minority or marginal (Alam, 2008; Arnett, 2008; Henrich et al., 2010; Alcalá & Cervera, 2022; Scheidecker et al., 2023a; Remorini, 2024) The science of ECD and the public interventions and policies that result from it in most countries around the world assume that human developmental potential is universal, regardless of the circumstances in which a person’s life unfolds (Remorini, 2024). The primary assumptions of the global ECD scientific and political movement are derived from the research findings in the fields of developmental psychology and neuroscience, which have been predominantly gathered from minority-world contexts and subsequently applied to the global population (Scheidecker et al., 2023a). This represents an “epistemic exclusion” (Scheidecker et al., 2023b) whose consequences at the theoretical, methodological, and practical levels are evident. This is because it ignores the research that does not readily fit into a predefined framework that excludes other cultural ways of understanding child development (Remorini, 2024).

Despite the growing recognition of cultural diversity as a crucial starting point for scientific research on child development, studies and theoretical approaches to child development and learning continue to exhibit a tendency toward culturally biased conceptualizations of normalcy (Remorini, 2024). This is evident in models of pedagogy, developmental milestones, and outcomes of learning processes, which are still largely based on what is expected of “average” or “typical” children. This is a consequence of the narrow scope of research evidence that is currently considered.

The goal of ECD research and interventions is clear: to enhance or optimize children’s development to reach their full potential, with a focus on neurodevelopment. These studies and interventions are often proclaimed as “based on the best available evidence”. Specifically, they are seeking to determine how environmental conditions influence brain structure and function, and to identify whether this is beneficial or detrimental to overall development (Scheidecker et al., 2023a). This is based on a very specific idea of the environment and how it affects us. It reduces the focus to one organ (the brain) and ignores the fact that humans are complex beings and cannot be reduced to their brain or genes. Furthermore, people’s lives cannot be reduced to a couple of socio-economic indicators or a pre-defined list of parenting practices. Practically speaking, this means intervening in maternal child-rearing practices—a key component of the environment—that are harmful or compromise normal and optimal neurodevelopment. The word “maternal” is emphasized here because it is the child’s biological mother who is the focus of targeted interventions. Other individuals and their actions, beliefs, and ways of interacting in the environment where the child grows up are ignored, despite their decisive impact on the child’s development. In this context, children and families in majority world communities are typically described and evaluated according to a predefined epistemic framework consisting of a set of measures, constructs, and theories derived from minority-world thinking (Scheidecker et al., 2023b). This raises the question of whether the assertion by scientists and policymakers that they are using “the best available evidence” is, in fact, accurate.

It is imperative that we expand our view of development beyond neurodevelopment and focus on the child as a whole organism existing within a specific environment. This approach must integrate disciplines like anthropology and cultural psychology that focus precisely on individuals developing in the context of dynamic interactions with their environments (Bronfenbrenner, 1987). In this regard, it is necessary, as Säljö (2020) suggests, to move toward a way of studying development that no longer treats the social and cultural world where people live as epiphenomenal. 

As we have been arguing for more than a decade, child development processes constitute a transdisciplinary field of study (Shonkoff & Phillips, 2000) to which ethnography contributes through its methodology and scale of analysis. Ethnography is interested in the cultural dimensions of human development and understands it as a situated process (Remorini, 2023, 2024; Weisner, 1996). The only way scholars can truly contribute to understanding the development of minoritized children, such as those who belong to so-called BILPOC communities (Black Indigenous Latinx and People of Color) is by demonstrating how culturally situated developmental goals and values guide children’s everyday experiences and their interactions in their specific environments.

Human development is a complex and multidimensional process. It involves people in their dynamic exchanges with the environments in which they live. These exchanges are multimodal and integrate vital functions and areas of behavior. In other words, cognitive, perceptual, motor, and affective functions are brought into play simultaneously in the activity of the person in their environment. They can be separated only for analytical or assessment purposes. For example, an assessment might determine whether a function is present or has reached expected maturity (according to the child’s age), or if training or education is required. Either of these possibilities involves establishing criteria and parameters. This is not an a-theoretical or an a-political process.

In recent years, there has been a growing interest in discourses on the importance of considering social and emotional skills—also called “soft skills”—along with cognitive and motor skills in learning. Children (pupils) must be trained in their development to achieve optimal outcomes in health, personal relationships, academic results, and future work careers. Ultimately, and according to “the best available evidence”, the development of these skills in childhood will, over time, allow them to become adults who are successful in life. This is usually measured in terms of academic performance and social achievement (Stafford-Brizard, 2017).

This “discovery”—the recognition of the value of social and emotional skills and the necessity of training these from childhood—opens a series of reflections relevant to anthropological (ethnographic) research in majority world societies. Building on this, ethnographic data collected between 2013 and 2018 in rural indigenous communities in Northwestern Argentina seek to provide a conversation based on these reflections.

## 2. The Role of the Environment in Child Development

Anthropological research definitively shows that human beings learn skills crucial for participation and competent performance in the environments in which they participate from infancy onward. It is not just the content of what we learn, but also how we learn—including the patterns of social and spatial organization and activities that enable such learning to occur—that has a central ontogenetic and phylogenetic value for our species (Remorini, 2023). The environment is therefore central to development. It is important to understand it as a combination of processes and phenomena that occur at different structural levels (Bronfenbrenner, 1987; Remorini, 2023) rather than as a set of variables, such as household income, mother’s level of education, access to services, or others, which tend to dominate certain types of discourse on ECD.

Studies carried out in Latin American indigenous societies have unequivocally demonstrated that children’s early contribution to domestic chores has a significant impact on their development of sophisticated social, cognitive, motor, and emotional skills (Remorini, 2023; Rogoff, 2003, 2014; Alcalá et al., 2014; de León, 2015). This research has also identified significant cultural variations in the sequence and timing of developmental milestones that do not align with the age expectations set by W.E.I.R.D models and screening tests that are applied without question. Furthermore, studies emphasize the necessity of observing children in their natural environments to gain insights into their developmental patterns.

From an ecological approach to human development, it is a fundamental assumption that development results from the dynamic participation of people in environments and activities, and in their progressive mutual transformation (Bronfenbrenner, 1987; Ingold, 2012). Bronfenbrenner (1987, p. 312) was clear: “development involves making the world one’s own and becoming a person in the process”. People are growing as dynamic entities who restructure their environments progressively. They can be considered becomings—a path to be traveled (Ingold, 2012). Each person’s path is constructed through a recognition of patterns, rhythms, and regularities in the environment, which, in turn, shape and are shaped by the path of others. Development is, therefore, a historical process rooted in the articulation of collective experiences and anchored in the passage of historical time (Ingold, 2000, 2011).

Developmental changes occur because of people actively engaging in routine activities that require the learning and practice of different skills (Rogoff, 2003; Ingold, 2011). Bronfenbrenner (1987) asserts that development occurs in two spheres: that of perception and that of action. Children perceive changes in their environment and perceive that their ways of relating to and participating in it change over time. This in turn modifies the environment and the development process. It is therefore crucial to identify and describe how specific forms of interaction and participation shape this mutual transformation. The activities in which children engage constitutes the microenvironment we need to describe for understanding this process. They demonstrate the genesis of emotional, motor, cognitive, and social skills, which achieve higher levels of complexity and sophistication as the environment poses new challenges. Children give meaning to their actions and those of others and elaborate new objectives because of a dynamic learning process.

This vision is based on the understanding that there are different possible and desirable development trajectories, shaped by the characteristics of the environment and sociohistorical time. It is curious that, despite recognizing today that each child starts from a different situation, which seems to challenge the linear and deterministic approaches of the last century, the points of arrival of the path are still presumed to be the same for all. In other words, we still think in terms of one single “typical” or “normal” trajectory (Remorini & Rowensztein, 2022). We must acknowledge not only the diversity of paths that can be followed, but also the multiplicity of potential outcomes and destinations. It is not enough to merely pursue different paths to achieve the same end (such as a certain level of academic performance, personal success, or a specific type of employment in adulthood). Each community defines these goals and, as a result, organizes the environments in which children grow up and develop in accordance with their cultural values, social organization, and changing historical circumstances (Ingold, 2011). They do not limit themselves to the narrow benchmarks established in W.E.I.R.D societies.

My ethnographic research shows that the development of so-called “socio-emotional” skills is a crucial component of the child-rearing practices and socio-cosmologies of certain communities (Remorini, 2015). However, it is a component that is often overlooked by dominant discourses on ECD, which propose different reasons for its importance.

In what follows, I will describe a cultural practice that promotes and trains skills in children, the care of livestock or *haciendita*, and discuss their impact on their personal development and social performance, drawing on basic notions of ecological approaches to human development and the concept of “mutual raising”, a concept applied by Bugallo & Tomasi (2012) to analyze human–livestock relationships in the Andean highlands, as kinship and care relationships.

The cultural knowledge, values, and practices regarding children’s learning and development I will describe here emphasizes reciprocity, respect, autonomy, and interdependence, and must be understood within the framework of ecological relationships between people and their environment. In this regard, I argue that it is necessary to situate interactions between children and the environments in which they participate within the framework of local cosmologies, as they define ontologies and appropriate relationships between humans and other living beings. In this sense, the diversity of cultural understandings about expectable ways of interaction between children and others (humans or not) is a central aspect to take into consideration when analyzing human development because these local understandings are the basis of cultural models about the course and development of life, having an impact on children’s daily experiences (Remorini, 2015).

## 3. Purpose and Methodology

### 3.1. Study Population

This article is based on a major ethnographic study on childrearing practices conducted in the Department of Molinos. The department is in the south of the province of Salta, Argentina, in the Northern Calchaquí Valleys region. This region has been inhabited for over 2500 years. Initially, it was settled by the *Diaguita* or *Calchaquí* people, who spoke the *Kakan* language. Other groups who spoke *Quechua* came here because of the Inca expansion and settled in the region in the fifteenth century. This resulted in a high degree of cultural homogeneity and social practices that are characteristic of the entire Andean region. The current population is a mixture of indigenous and Hispanic elements. The population is distributed in a group of scattered settlements and large private farms that clearly reflect a type of latifundista exploitation. The last census shows that the department has a total population of 5565 inhabitants. Of these, 3500 (62% of the total) live in rural areas and 3984 (71% of the total) are children under 12 years of age (INDEC, 2010).

The town of Molinos (locally called *el bajo*) is where the administrative and service activities—education, health, and commerce—take place. Meanwhile, the farms (*el alto* or *los cerros*) focus on agricultural–livestock activity for commercial purposes, for personal consumption, and handicraft and wine production. The economy of the valleys is based on the use of local resources, production, and consumption on a domestic scale. It is linked to different productive and commercial modalities on a local and regional scale. This economy is based on a special material and symbolic relationship between the communities and their environment (Göbel, 2002).

The ranches (*fincas*) are vast tracts of land that have belonged for generations to the same family of landowners. This legacy can be traced back to colonial times, although in the last two decades, some have been acquired by foreign capital. The town and the ranches present a semi-urban structure. This is characterized by agrarian villages associated with the arable fields and extended families, including those in domestic labor, under a paternalistic regime, in the agricultural estates. Households are dedicated to agricultural work and the care of livestock (cows, goats, and sheep) for subsistence and self-consumption. Women and children of both genders participate in this activity. The household (*la casa*) constitutes the basis of production, consumption, and sociability. People are strongly rooted in their local communities. The families in the different sectors of Molinos are linked by kinship ties and some of them have dwellings in both the *fincas* and the town. This allows them to optimize available resources in each zone (Remorini et al., 2019).

Households differ in terms of their composition and size. The village is home to a majority of nuclear or vertically extended families, with an average of four children. In contrast, ranches are characterized by vertically and horizontally extended families, matrilocality, and an average of seven to eight children. They are relatively autonomous in terms of production and resource management, but they maintain parental ties of reciprocity and cooperation with individuals and families beyond the boundaries between village and ranch. These are clearly evident in the joint performance of subsistence activities and ritual celebrations that are carried out annually.

Regarding health services, Molinos has a first-level provincial hospital located in the town and six health posts on the ranches, run by nurses and/or health agents who carry out primary healthcare activities. In relation to the school, the houses in the ranch where we have worked are located between one- and three-hours walking distance from the nearest establishment. This has led families to adopt different strategies to ensure their children can attend school. Some children in kindergarten are housed at the school from Monday to Friday, while other families move to homes near the schools during the school year. These strategies ensure that children can continue their schooling from the initial level. However, they have had an impact on children’s participation in traditional domestic activities with their family, such as the care and maintenance of the livestock, which rely on this type of collaboration.

The inhabitants of the *fincas* have a strong bond with their animals (*haciendita*). The daily routines are organized around their care. Times, spaces, and tasks are distributed among relatives and neighbors. Relationships of cooperation, exchange, and complementarity ensure the increase in and maintenance of the herds, as well as their reproduction and perpetuation (*multiplico*) through inheritance to younger generations (Remorini et al., 2019; Desperés & Remorini, 2022).

### 3.2. Objective, Data Collection, and Analysis

This article focuses on children’s activities that involve a close and daily relationship with the livestock. It proposes to demonstrate how this relationship plays a central role in the development of social and emotional skills that are of ecological value, in the sense that they are key to the development of children in this environment.

The research from which this article is derived follows an ethnographic methodology that is suitable for studying activities and relationships that occur at the microlevel. This scale makes it possible to identify the processes involved in children’s development in their immediate environment, and to focus on the perspectives of the people being studied. Throughout this research, we used different ethnographic techniques in a complementary way, including direct and participant observations of behaviors and interactions within the framework of household-scale routines, as well as participation in various community activities. This is a standard practice in ethnographic work that is known for its long duration, cohabitation, and successive stays over time. Furthermore, we conducted in-depth interviews and informal conversations during these activities.

We observed children’s participation in different subsistence activities, including the care of other people and animals, in a systematic and participant manner. These observations were conducted in several stages. We began with unstructured observations of the day’s life in a small number of households from the town (*pueblo* or *el bajo*) and the farms (*fincas* or *el alto*). We observed them for most of the day, for an average of 10 consecutive days. We also accompanied children and other people on their daily journeys through different spaces, following their movements as they engaged in various activities, including play, visits, and errands. In the second stage, we conducted systematic and structured observations at two-hour intervals in a larger number of households. We focused on children’s behavior and their interactions with other children and adults. An instrument was designed to collect and compare information on different aspects of parenting. We also made spot observations during visits to different families for interviews and other purposes. We recorded these observations in field notes and structured observation grids, video, and photographs to document the activities.

While we prioritize observation, it is essential to recognize that people’s knowledge and categories play a vital role in understanding the activities and behaviors that are observed. We concur with Gaskins (2000) that importing cultural bias into our understanding of what children’s activities are most important to adults who care for them is a risk we must avoid. Based on observations and video records, we conducted interviews with adults and children to gain insights into childcare and child-rearing practices.

Through a selection of interviews, field notes, and audio–visual records collected between 2013 and 2018 (as part of a larger research project spanning a total of 10 years), we focused on describing and analyzing the ways in which the environment facilitates and sustains the deployment of crucial skills for children, which enable them to engage in responsible caring for others, in this case, the animals that comprise the *hacienda* (livestock).

Throughout the entire research process, data analysis followed the grounded theory procedure (Glaser & Strauss, 1967; Cresswell, 1998), and Nvivo 11 (QSR International©), which provides tools that allow the classification, selection, and codification of textual references, hierarchizing them, identifying and linking different local categories of analysis, and inductively elaborating hypotheses based on the links between categories.

This software was used to construct a tree of nodes for the general project, of which this paper focuses on a particular aspect of child rearing and child participation. In this global coding scheme, a sub-node, “Relationship with the livestock”, was derived from the mother node, “Child Participation in Domestic Activities”, which in turn was divided into five third-level sub-nodes, “Origin of the Hacienda”, “Property Relationship”, “Daily Life with the Animals”, “Luck”, and “Rituals”, which include community celebrations within the annual cycle, such as “*Señalada*” and “*Corridas*” (fourth-level sub-nodes). Within the general node and according to the specificities of each sub-node, the verbal references or observational records related to the processes and arrangements related to the acquisition, reproduction, and maintenance of the farm were categorized and analyzed, identifying the different local categories and their semantic scope in the discourse, their horizontal and vertical relationships. This analysis was applied specifically to 8 semi-structured interviews with caregivers with whom we dealt with the subject, 4 audio recordings of informal conversations with children, and several excerpts from field notes where scenes of children’s intervention in activities related to animals were observed and recorded.

### 3.3. Ethical Safeguards

All participants provided their free and informed consent to participate in the research. Their personal data have been safeguarded according to national regulations and the studies have been evaluated by the ethics committees of the Catholic University of Salta and the National University of La Plata (Argentina) and in accordance with national law 25.326 on personal data protection.

## 4. Results

### 4.1. Mutual Raising: Autonomy, Interdependence, and Responsibility

“People do not mourn for a small child, for a small child is not a person (ran). When he tethers the cattle and herds the goats, he is a person. […] A man will not say that he has a son till the child is about six years of age. A small child is buried by old women and without sacrifice” (Evans-Pritchard, 1956, p. 146).

The results of this research show how children are initiated in some activities of cultural value—such as raising other children and domestic animals—and how they exercise different skills that enable them to take different responsibilities in the care of others (human and non-human). They also show how children show their preferences and interests to be involved in certain tasks. We will account for the active role of children in creating their own learning environments and for the role of the environment in supporting and receptively engaging with children’s initiative and autonomy in learning and performing these care tasks.

It is well documented that personhood is not a given fact in numerous American indigenous communities. Rather, it is related to the capacity for productive sociality (Murray & Tizzoni, 2022), which requires effort on the part of the individual. Becoming a person implies a path in which the interactions between children and their environment are of central importance. Caregivers must facilitate this path, while carefully observing and recording what signs children show of moving in the direction of expected and valued forms of sociability (García, 2015).

Curiosity and interest in engaging in interactions and activities is the first step in moving toward autonomy within the framework of interdependent relationships between people and other entities in the environment (Remorini, 2015, 2023). They are driven by a desire to learn (*les nace*). This can be seen in the case of children who take on the responsibility of raising their livestock.

Children’s initiative and motivation is recognized and respected by adults, who in turn seek to create opportunities for children to become involved with animals:

My brother has horses and when he can my brother saddles up a horse and she (his daughter) sits down and you can’t get her down and she likes to ride on the horse by herself, the other day I told her dad ’don’t let her sit alone’ but she wants to get on the horse so at least for a while she is with the horses, her dad wants to help her but he says ’the bow is mine’.

(ID 287, female, 28 years old, Gualfin 2016)

During the lunch at M’s house, it was observed that one of the children, aged six, was attempting to persuade a goat to enter the pen. M indicates that he is engaged in training activities in anticipation of their imminent departure for the hill to “round up the cows”. The other children observe and laugh as the boy fails to achieve his goal. M advises children to “stop bothering” him. I inquire as to whether the child enjoys interacting with the animals and assisting with their care. He responds that this child is willing to do so, but that others are less enthusiastic about the task.

(Fieldnotes, Gualfin, 2018)

As Fernando García (2015) points out in his study in another region of the Andes “the development of autonomy also entails the development of children’s initiative… Adults encourage the development of children’s initiatives when they consist of ‘doing’ something to contribute to the success of the activity… The formation of a fully developed person who displays the qualities of ‘being people’ focuses on the cultivation of personal autonomy not only for participating and learning in pursuit of personal interests within the family’s everyday activities but also for personal growth depending on his or her particular capabilities” (2015: 143).

### 4.2. Cuidar la Haciendita: Children’s Engagement in the Care of Animals

For a child to be involved in this activity, it is necessary that they have reached a high degree of autonomy since this task requires knowing the environment, moving in space, following instructions, improvising, assessing changing conditions, handling the needs of the animals, and cooperating with others. The children’s chronological and social age determines their eligibility to participate in this activity. Those who are considered *guaguas*—i.e., babies or infants who are just starting to walk freely and are still highly dependent on adults—are excluded from this activity. Instead, children who do participate in this activity are typically five years of age or older. They are in the middle childhood stage (Weisner, 1984), which is when they typically engage in more active and responsible roles in their communities.

To analyze the participation of children in the care of livestock, we must first describe how they are able to do it and what role the social environment plays in facilitating and encouraging this participation.

In the characterization of the study area, we have previously distinguished between two sectors: the town and the farms. The description presented here comes from the latter sector, since the town households currently have fewer opportunities to have corrals and cultivable land, due to the increasing urbanization of areas called *orillas* (outskirts), where, until two decades ago, a rural system of land use prevailed. Many households in the village keep livestock *en el alto* (in the farms) through cooperative and reciprocal arrangements with relatives and neighbors that are maintained over generations. This fact prevents children from becoming involved in the care of these animals.

The situation on the farms is, in fact, very different. The daily bond between boys and girls with their *haciendita* begins at an early age. Boys and girls work together on the family farm, helping with agricultural chores and gathering fruits, firewood, and medicinal plants, while also participating in animal herding. These tasks are conducted in collaboration with other members of the household. Adults use the expression “*se crían con la hacienda*” (they grow up with the animals) to mean that, from an early age, they observe, accompany, or take part in various tasks related to its care. In the first years of life, this participation is peripheral (Rogoff, 2014; de León, 2015). They are brought or carried on the back (carried by women and girls in shawls) until around 4–5 years of age, when a closer interaction with the animals begins to develop (Desperés & Remorini, 2022).

It is a fact that not all children are interested in this type of activity. Some children prefer to stay at home with the women of the family, helping to care for the *guaguas*—whether they are their siblings, cousins, or nephews and nieces. Other children are much more interested in gardening.

Those interested in livestock form a strong emotional bond with some of the animals in the house, which adults call affliction. This means they are aware of what may happen to them (illnesses, death, giving birth, rejection of the offspring, etc.) and they take action to ensure their wellbeing. On the contrary, there are boys and girls who *no se afligen* (do not grieve) or *se olvidan* (forget) their animals. Concretely, in a family, there might be children who take care of animals because *les nace* (they are naturally driven to) and others who do it at the request of the elders. The children in these households gain a keen understanding of their animals through daily interactions: they are able to recognize distinctive physical and behavioral traits, and often name the animals based on their observations. They quickly identify each animal’s unique personality and take precautions when approaching or handling them. Fieldwork observations exemplify this:

There are several goats and kids in Doña Fermina’s corral. T enters and shows me the newborn goats. A girl strides purposefully past the goat, calf in tow, and surveys the scene. With a decisive motion, she moves on, skirting the group until she reaches the next set of goats. She lifts a calf and brings it to me near the entrance to the corral, clearly indicating that I may enter. I enter. In the afternoon, I told her mother about this behavior. She confirmed that some animals are docile while others are aggressive. She explained that the most aggressive one has hit her with her head and doesn’t allow contact, which is why children avoid it.

(Fieldnotes, Gualfin, 2018)

A and AN (two siblings, female and male) show me some videos they filmed with their cell phone when they accompanied their family to the corridas on the hills. A proudly declares: “That’s my cousin who is caping a bull”. This is my cow. This bull is called “*Coloradito*” (Researcher: and these other cows are yours?) Yes. That one is the son of that one. That one is the daughter of another. The mother of that one is the daughter of another that has died (pointing to the animals on the screen).

(Fieldnotes, Gualfin, 2018)

### 4.3. Criar y Multiplicar: How to Become a Shepherd from Early Infancy

Initiation into animal breeding is favored by the inheritance of animals at different moments of their life trajectory, many of which have a ritual expression. Children receive animals *en suerte* from birth and during baptism or the first haircut (*ruti*) from their relatives, especially grandparents. This is a rite of passage that is widely observed in the Andean region. It marks the child’s introduction to society and incorporation into their community and the networks of relationships that constitute it. Girls and boys also become possessors of goods in the form of money, animals, and/or land from this moment on (Desperés & Remorini, 2022).

It is said that girls and boys receive animals *en suerte* (literally translated as to have luck) (Bugallo & Tomasi, 2012), and they must keep them, raise them, reproduce them, consume them, and eventually exchange them for other animals and material goods throughout their life trajectory. Other family members care for these animals until the children are ready to take responsibility for them. It does not matter how much is received. The goal is to maintain and increase the herd. This is where the prestige and status of people in these communities lies. It depends on luck whether girls and boys can “make” more animals, that is, reproduce and perpetuate the herd. *Suerte* is not the same for everyone:

(Researcher: “And how do you do it? How do you give them out?”) “When they’re little, I give one to each of them, and if they’re lucky, they keep reproducing and reproducing. But the ones who aren’t lucky don’t have any, and theirs die, or they get sick, or they don’t have kids. But there are others who are lucky…”

(ID272, female, 45 years old, Gualfín, 2018)

(Researcher: “Could it be because he takes better care of them? Because he pays more attention to them?”) “Maybe luck follows him (*la suerte lo sigue a él*); some people are just lucky. I used to have cows. From just one cow, I had five cows. And out of those five cows, I ended up without even one.”

(ID299, female, 42 years old, Gualfín, 2017)

What does luck consist of? Some studies in the Andean region define luck as a vital energy that allows life to reproduce and regenerate, developed through the human–animal relationship (Bugallo & Tomasi, 2012). In our data, it is usually used in at least three senses: 1: to refer to a characteristic, quality, or condition that is inherent to the person from birth, 2: as a condition that can be found in both an animal and a person, and 3: as the result of a lifelong relationship between man and animal. Therefore, there can be different situations: children who did not have luck can find it in certain animals they acquire during their lives; some people can pass their luck to animals, so that when they are slaughtered or sold, their luck goes with them; also, attempts can be made to increase luck through rituals performed by traditional specialists.

Most importantly, through rituals performed at key moments in the annual cycle, luck must be taken care of. The livestock is part of the home, of the family, so it requires as much care and respect as the rest of its members, although, unlike people, the livestock can eventually be sold or slaughtered for consumption or sale. In these situations, however, there are rules that must be followed for this to be performed respectfully.

Children, as the owners of the animals, exercise their right to keep them or to exchange some or all for needed money to acquire something that they or their families need. This decision is respected by their parents although they generally advise them not to do that, which means that herds reach hundreds of animals, which are inherited and proudly displayed as the result of work sustained over time by family groups.

### 4.4. Is la Suerte a Cultural Manifestation of Mutuality of Raising?

The research conducted in Andean communities (Bugallo & Tomasi, 2012; Pazzarelli, 2014) makes it clear that when people and animals interact in certain ways, it creates an affinity. This affinity is something that must be taken care of, as mistreating the animals or not following certain culturally established practices and techniques (in terms of their care and even slaughter) can result in a loss of *suerte*—for the person, the animal, or both. The non-respectful treatment of animals, a lack of interest, or carelessness are attitudes that jeopardize this relationship of affinity that is expressed in the concept of *suerte* (luck).

The concept of “mutual raising” (Bugallo & Tomasi, 2012) accounts for these forms of relationships between people and animals as relationships of kinship and mutuality, as opposed to the unilateral relationships of Western domestication. In these Andean communities, the human–nature or social world–natural world dichotomy does not exist. All beings raise and are raised by sharing food, care, affection, recognition, mutual aid, and affliction. In the framework of this relationship of mutual raising, *la suerte* will transform any animal, in one possible to be bred and a human being in a shepherd (Pazzarelli, 2017).

Pazzarelli (2014) states that *suerte* is a relational force that allows one to become a shepherd. This activity and relationship allow the development of a unique set of knowledge, skills, and dispositions that are not shared with those who choose other paths. This enables them to gain knowledge and experience of different spaces, understand animal morphology and physiology (especially related to feeding, reproduction and health), calculate risks, and make decisions in a way that differs from those whose trajectory is linked to other types of social relationships, activities, and spaces.

Taking luck into account highlights at least two important aspects for understanding the development of children in these communities. First, we must consider the affective dimension that emerges in animal care. The demonstrations of affectivity and interest toward the animals, given that they are considered part of the group and of the house and the family, unequivocally demonstrate that when the *haciendita* is subjected to harm or damage, this gives rise to experiences of suffering, such as *susto* (fright) and *pena* (grief). These are emotional and physical states that originate because of traumatic, stressful, or unexpected experiences. The second point concerns the capacity for agency that children develop over time. Their agency is strengthened by luck. Thanks to this agentive capacity, they are able to “make animals” (Medrano, 2016), which means naming them, taking care of them, marking them, vaccinating them, perpetuating them, and other actions that integrate them into the family and the home.

The concept of *suerte*, as evidenced in the local narrative, illustrates the interdependence of animals and humans from an early age, underscoring the importance of nurturing these relationships throughout life. For children aspiring to become shepherds, *suerte* serves as a pivotal factor in their development, enabling them to perpetuate a legacy and contribute to the sustenance and prestige of their families. As they develop the ability to “make animals”, children also become shepherds, a valued identity in this rural community way of life.

## 5. Discussion—Care for Others Is a Developmental Milestone

The process of becoming a shepherd underscores the necessity for children to develop a set of social, cognitive, motor, and affective skills that enable them to engage and interact with their environment in a manner that is both distinct and appropriate. These skills play a significant role in the formation of an individual’s life trajectory and cultural identity, among other potential outcomes, within this particular environment and in other contexts that exhibit similar characteristics. These skills facilitate the development of autonomous individuals, who nevertheless remain closely interdependent with their environment.

Following García (2015), children’s autonomy is not a process undertaken by a person free of social ties; on the contrary, it is the development of a person rooted in a broad network of relationships, which include, in the case of Andean households, people, animals, and other non-human entities. Children activities should be aligned with the wellbeing of others, and, in that sense, should be respectful to others. Adults, in turn, should respect children’s autonomy (Remorini, 2015). The balance between autonomy and interdependence and, within it, the progressive responsibility for caring for others (animals or people) synthesizes a goal pursued by childrearing in these communities. In this framework, becoming a caregiver is a milestone of development and is possible thanks to the development of the skills described above. This leads us to raise the need to study these milestones from the community’s perspective, as they may not be the same and may not be aligned with the conventional norms or expectations associated with the “typical” or “normal” milestones as defined within the context of the W.E.I.R.D. models.

As Bronfenbrenner (1987) has stated, the routine activities in which children engage represent the “environmental facts” that exert the most significant influence on their development, thereby establishing favorable contexts for the study of human development. Anthropological (ethnographic) research has focused on activities as micro-environments of development, with the objective of studying and understanding the processes of enskillment (Bronfenbrenner, 1987; Ingold, 2000). The findings of our study enable us to engage in constructive discourse with other research projects that emphasize the significance of autonomous learning through active involvement in activities that are of value to the community (Rogoff, 2014; Alcalá et al., 2014; de León, 2015).

As observed by Murray and Tizzoni (2022), in the case of the Mapuche of Chile, children are included in the ongoing efforts of their families and communities. They are treated as regular participants in the community-based organization. They share the same expectations and opportunities to contribute as all others, according to their interests and abilities (Rogoff, 2014; Alonqueo Boudon et al., 2023). In this context, children are expected to participate in and are incorporated into community activities from an early age. Through these activities, children develop their ability to initiate actions within social life, which strengthens their sense of will and volition, as well as motivation, or the driving force of their actions (de León, 2015; García, 2015; Remorini, 2023; Alonqueo Boudon et al., 2023).

By “sophisticated and complex skills”, we refer to a set of skills that includes, in the case analyzed in this article, the following: the ability to observe and listen attentively to multiple events occurring simultaneously, a broad listening that includes not only people but also environmental events that configure specific signals to carry out an activity. Spatial orientation and the capacity to calculate distances and interpret changing environmental information are also crucial, as well as solving problems, coordinate actions with others, and learn specific vocabulary. Additionally, it is important to understand and anticipate the physiological needs of other beings, such as animals. Motor skills are also essential for the care of animals, including handling a variable number of animals during their displacement, as well as the ability to gather, redirect, and lead them to safe places. Finally, emotional commitment to the care of the farm is a crucial aspect of the role.

The observation of children’s daily routines allows for the recognition of how children simultaneously employ a range of skills in the completion of various tasks, whether in collaboration with others or independently. To illustrate this, one task that is frequently assigned to them when they are not at school is “taking out the livestock” (*sacar al haciendita*). This entails removing the animals from their pens in the morning, leading them through pastures, ensuring they consume sufficient food, returning with all the animals in the late afternoon, and returning them to their pens. This often involves the use of leather lassoes to “*atajar los animals que se escapan*” (catch animals that scatter) which implies displaying physical strength and dexterity. It is also necessary to make sure that they do not eat other neighbors’ pastures or mix with other herds.

It is only by bringing all these skills into play, either simultaneously or in a progressive manner, as one gains proficiency in the activity, that one can “make animals” and become a shepherd, multiply the livestock, and thereby contribute to the growth of the family and the household.

By drawing on Ingold’s (2000) ideas about “inhabiting an environment”, one can see how the world emerges and becomes meaningful for those who live in it, as it is incorporated into a regular pattern of activity. Human life trajectories are shaped by this embeddedness and close relationships with diverse forms of life, as well as by a practical engagement with a set of activities considered to be vital. In summary, animals and children are “made” in an environment that is designed through their interactions, and the skills that result from this process of “mutual raising” distinguish their trajectories from those of other children.

From the perspective of the Molinos families, the care of the *haciendita* and younger children is a responsibility that is bestowed upon them when they have demonstrated an adequate level of autonomy. This autonomy is assessed based on the child’s ability to assume responsibility for the aforementioned beings for a designated period of time or in an activity that aligns with their capabilities. In other words, there are multiple livestock care tasks that children can perform according to their age and level of competency. As articulated by a mother interviewed for this study, “I would never assign responsibility to my child for a task that I believe his is not prepared to undertake”.

At this juncture, the pivotal role of the social context becomes evident. It serves to either facilitate or restrict, as deemed appropriate, based on an evaluation of the learning potential and associated risks inherent to the activity in question. It is tasked with fostering children’s attention toward pertinent events and activities, while respecting their autonomy and initiative. However, this is conducted in a manner that provides careful guidance, allowing children to discover “their own way” (Ingold, 2007) and positioning them as active agents of learning: “children are involved as much as their teachers, as active and creative participants in the learning process. They participate by making their own contributions to shaping the contexts in which learning occurs and knowledge is generated” (Ingold, 2007, p. 113).

The activities of caring for animals and other people are a central part of everyday life in Latin American indigenous societies. In other studies (Remorini, 2009, 2011, 2015), we have demonstrated the role of children in caring for other children and older family members as part of cooperative and reciprocal relationships at the familial and community levels. All individuals engage in caregiving and receive care, and these polyadic relationships are shaped by moral obligation, respect, and affection.

Based on the findings of this research and those of previous studies, our hypothesis can be stated as follows: caring for others represents a crucial milestone in child development. This is because it implies the achievement of maturity in the development of a set of skills. Consequently, in some societies, a child is only regarded as a “person” once they have demonstrated the capacity to care for others, as evidenced by Evans Pritchard’s aforementioned quote. The deployment of socio-emotional skills is a prerequisite for caring for others.

In many cultural contexts, children are excluded from such tasks, which are instead reserved for adults. This segregation is evident in the prioritization of activities considered proper to children, such as play, and the segregation of children from productive activities or those that imply a contribution to the household (Rogoff, 2003; Alcalá et al., 2014). In contexts where the spatial and social segregation of children are the norm, other competencies, frequently assessed and evaluated on an individual basis, are often prioritized. In this regard, a substantial body of research has demonstrated that children from indigenous societies who are trained in a range of activities, including horticulture, gathering, hunting, animal husbandry, and construction, exhibit a remarkable capacity to acquire and apply a diverse set of skills that are transferable to other domains of social life (Maynard, 2022; Miller, 2006; Morelli, 2012; Wyndham, 2010; Lorente Fernández, 2015; Ng’asike, 2010; Remorini, 2023; Cervera Montejano, 2023; de León, 2023). Such abilities are not typically considered in academic or other contexts where children’s knowledge and competencies are evaluated. Conversely, the activities that are central to children’s lives are often minimized or underestimated.

## 6. Conclusions

In this paper, we propose to account for this complex social and emotional network between children and their animals, by showing how the so-called “socio-emotional” or “soft skills” from contemporary positions and theories of learning are the basis for the upbringing and sociability of girls and boys in Latin American indigenous societies. These skills are not considered less relevant than other skills, such as memory, attention, orientation, or other executive functions; rather, they are integrated with them. These skills are fundamental to the concept of “personal and social success”, which is based on a distinct understanding of the individual, life trajectory, and social relations. This differs from the assumptions made in W.E.I.R.D approaches to child development.

In majority world communities, such as the one described here, autonomy in interdependence, mutual care, and a respect for all forms of life are cultural values that shape children’s developmental trajectories. As demonstrated in previous research (Remorini, 2015, 2023; Desperés & Remorini, 2022), children are encouraged from an early age to direct their attention toward events and situations in their environment that require observation, comprehension, and active engagement. This includes respect and care for other forms of life, including non-human ones. In the context of animal husbandry, this is evident in the manner in which we interact with them, the affection they display when being spoken to, handled, and fed, as well as in each of the aforementioned instances. Furthermore, it is evident that they experience grief *(se afligen, se apenan, se asustan)* when their animals become ill or die.

This close interdependence can be understood from the concept of “mutual raising”, which has a tangible expression in the “luck” (*suerte*) each person demonstrates by becoming an animal breeder and expanding their livestock, thus contributing to their personal development and that of their family.

By examining interactions between children and animals, it is possible to identify the skills and dispositions that are simultaneously engaged, resulting in distinctive developmental trajectories. The epistemological framework of indigenous socio-cosmologies contrasts with the analytical fragmentation and dichotomization that is prevalent in most scientific practices. This framework provides a basis for understanding childhood development and learning, as well as other domains of cultural life, through the lens of integrated functions. Over the centuries, we have learned to separate, isolate, describe, and delve deeply into various aspects of development. We tend to compartmentalize development into distinct behavioral domains, viewing the child through the lens of our instruments, and segmented families and environments into a limited set of indicators that correlate with a potential risk of developmental deficits.

As highlighted by the Ecology of Human Development and as documented by several papers on these societies already referenced in this article, it is impossible to consider the concept of development without acknowledging the local theories that posit it as a path whose trajectory cannot be fully understood on an individual scale. Similarly, the practice of isolating a child to evaluate his or her individual performance in a set of pre-established, decontextualized tasks often results in an incomplete and potentially distorted understanding of the child’s competencies. The utilization of tests in experimental settings can serve as a valuable tool for initiating inquiries or formulating hypotheses. However, it is imperative to recognize that such an approach should not be regarded as a singular and exhaustive methodology.

The diversification of methodologies pertaining to human development and the associated disciplines is a welcome phenomenon. It would be remiss to overlook the necessity of taking several additional steps. There is still a need to reconcile and agree on the limitations of theoretical and disciplinary approaches to child development. This will help to avoid the pitfalls of “methodocentrism” (Weisner, 1996) and “ethnocentrism” when studying and approaching child development in a practical way (Henrich et al., 2010; Scheidecker et al., 2023a; Remorini, 2024).

The comprehensive, microscale, ethnographic account of children’s everyday life offers insights into the significance of interactions and forms of participation in children’s developmental paths, as exemplified in the descriptions presented here. These insights are particularly relevant in understanding the early stages of children’s development within a specific context and historical moment. This description contributes to the framework of the interdisciplinary study of human development, providing support for debates both within and beyond the academic community. Local studies, employing methodologies attuned to specific contexts, based on observation and participation by the researcher, yield data and insights that we believe can inform discourse on children and their potential for participation in diverse environments, acknowledging their varied experiences, capabilities, perspectives, and interests. From an interdisciplinary perspective, employing a combination of methods and an intercultural approach that transcends ethnocentric biases, we can develop more effective proposals for interventions and programs focused on early childhood development.

## Data Availability

The data supporting reported results, generated during the study, can be found on databases available at Laboratorio de Investigaciones en Etnografía Aplicada (LINEA), Universidad Nacional de La Plata, Argentina, whose director is Dra. Laura Teves.
